# Supplementary material for: Mechanistic origins of temperature scaling in the early embryonic cell cycle
Source: Nat Commun. 2025 Aug 28;16:8045. doi: 10.1038/s41467-025-62918-0 (PMC12394406; doi:10.1038/s41467-025-62918-0)
Supplement: Supplementary file 3 — Description of Additional Supplementary Files [file 41467_2025_62918_MOESM3_ESM.pdf]

**Description of Additional Supplementary Files**

File Name: Supplementary Movie 1
Description: Movie of developing *Xenopus laevis* embryos at 21 ºC at 10fps. (related to Fig. 1)
*Xenopus laevis* eggs were fertilized and imaged in a temperature-controlled chamber by time-lapse microscopy (Fig. 1A). We then analyzed the movie to visually identify various early developmental events and characterize their timing (Fig. 1).

File Name: Supplementary Movie 2
Description: Movie of developing *Xenopus laevis* embryos at 11 ºC at 10fps. (related to Fig. 1)
*Xenopus laevis* eggs were fertilized and imaged in a temperature-controlled chamber by time-lapse microscopy (Fig. 1A). We then analyzed the movie to visually identify various early developmental events and characterize their timing (Fig. 1).

File Name: Supplementary Movie 3
Description: Movie of developing *Xenopus tropicalis* embryos at 21 ºC at 5fps. (related to Fig. 2)
*Xenopus tropicalis* eggs were fertilized and imaged in a temperature-controlled chamber by time-lapse microscopy (Fig. 1A). We then analyzed the movie to visually identify various early developmental events and characterize their timing (Fig. 2)

File Name: Supplementary Movie 4
Description: Movie of developing *Xenopus tropicalis* embryos at 26 ºC at 10fps. (related to Fig. 2)
*Xenopus tropicalis* eggs were fertilized and imaged in a temperature-controlled chamber by time-lapse microscopy (Fig. 1A). We then analyzed the movie to visually identify various early developmental events and characterize their timing (Fig. 2).

File Name: Supplementary Movie 5
Description: Movie of developing *Danio rerio* embryos at 20 ºC at 5fps. (related to Fig. 2)
*Danio rerio* were imaged in a temperature-controlled chamber by time-lapse microscopy (Fig. 1A). We then analyzed the movie to visually identify various early developmental events and characterize their timing (Fig. 2).

File Name: Supplementary Movie 6
Description: Movie of developing *Danio rerio* embryos at 30 ºC at 10fps. (related to Fig. 2)
*Danio rerio* were imaged in a temperature-controlled chamber by time-lapse microscopy (Fig. 1A). We then analyzed the movie to visually identify various early developmental events and characterize their timing (Fig. 2).

File Name: Supplementary Movie 7
Description: Movie of cell cycle oscillations in individual frog egg extract droplets across a temperature gradient (related to Fig. 4)
Time-lapse video showing oscillations in Cdk1 activity as measured by a FRET-based sensor in encapsulated *Xenopus* egg extract droplets situated within a temperaturecontrolled imaging chamber (see Fig. 4A). Each droplet contains cycling extracts and exhibits periodic changes in FRET ratio. Droplets are arranged along a spatial temperature gradient spanning approximately 12°C to 32°C.
