# Supplementary material for: Mechanistic origins of temperature scaling in the early embryonic cell cycle
Source: Nat Commun. 2025 Aug 28;16:8045. doi: 10.1038/s41467-025-62918-0 (PMC12394406; doi:10.1038/s41467-025-62918-0)
Supplement: Supplementary file 11 — Reporting Summary [file 41467_2025_62918_MOESM11_ESM.pdf]

nature portfolio

Corresponding author(s): Lendert Gelens

Last updated by author(s): July 17, 2025

Reporting Summary

Nature Portfolio wishes to improve the reproducibility of the work that we publish. This form provides structure for consistency and transparency in reporting. For further information on Nature Portfolio policies, see our Editorial Policies and the Editorial Policy Checklist.

Statistics

For all statistical analyses, confirm that the following items are present in the figure legend, table legend, main text, or Methods section.

n/a	Confirmed	
☐	☒	The exact sample size (*n*) for each experimental group/condition, given as a discrete number and unit of measurement
☐	☒	A statement on whether measurements were taken from distinct samples or whether the same sample was measured repeatedly
☒	☐	The statistical test(s) used AND whether they are one- or two-sided<br>*Only common tests should be described solely by name; describe more complex techniques in the Methods section.*
☒	☐	A description of all covariates tested
☒	☐	A description of any assumptions or corrections, such as tests of normality and adjustment for multiple comparisons
☐	☒	A full description of the statistical parameters including central tendency (e.g. means) or other basic estimates (e.g. regression coefficient) AND variation (e.g. standard deviation) or associated estimates of uncertainty (e.g. confidence intervals)
☒	☐	For null hypothesis testing, the test statistic (e.g. *F*, *t*, *r*) with confidence intervals, effect sizes, degrees of freedom and *P* value noted<br>*Give P values as exact values whenever suitable.*
☐	☒	For Bayesian analysis, information on the choice of priors and Markov chain Monte Carlo settings
☒	☐	For hierarchical and complex designs, identification of the appropriate level for tests and full reporting of outcomes
☒	☐	Estimates of effect sizes (e.g. Cohen's *d*, Pearson's *r*), indicating how they were calculated

Our web collection on statistics for biologists contains articles on many of the points above.

Software and code

Policy information about availability of computer code

Data collection	doi: 10.5281/zenodo.15591678 ; https://gitlab.kuleuven.be/gelenslab/publications/temperature_scaling
Data analysis	10.5281/zenodo.16269314; https://gitlab.kuleuven.be/gelenslab/publications/temperature_scaling; https://github.com/YangLab-um/temperature; https://github.com/YangLab-um/dropletDataProcessing

For manuscripts utilizing custom algorithms or software that are central to the research but not yet described in published literature, software must be made available to editors and reviewers. We strongly encourage code deposition in a community repository (e.g. GitHub). See the Nature Portfolio guidelines for submitting code & software for further information.

Data

Policy information about availability of data

All manuscripts must include a data availability statement. This statement should provide the following information, where applicable:

- Accession codes, unique identifiers, or web links for publicly available datasets
- A description of any restrictions on data availability
- For clinical datasets or third party data, please ensure that the statement adheres to our policy

Data Availability
Source data are provided with this paper:

- The western blot and droplet tracking datasets have been deposited in a Zenodo repository (doi: 10.5281/zenodo.15591678), which is publicly available as of the date of publication.
- All datasets necessary to reproduce the figures in the manuscript, along with the figure-generation code, are available at the Gelens Lab GitLab repository (https://gitlab.kuleuven.be/gelenslab/publications/temperature_scaling), and are publicly accessible.

Code Availability
Code is provided with this paper:
- All codes have been deposited in a Zenodo repository (doi: 10.5281/zenodo.16269314), which is publicly available as of the date of publication.
- All figure-related code, together with the necessary datasets, is also available at the Gelens Lab GitLab repository (https://gitlab.kuleuven.be/gelenslab/publications/temperature_scaling), and is publicly accessible as of the date of publication.
- The original modeling code used in this study is also included in the Gelens Lab GitLab repository.
- Code for image processing and analysis methods is available at the following GitHub repositories and GitHub repositories (https://github.com/YangLab-um/temperature; https://github.com/YangLab-um/dropletDataProcessing), publicly accessible as of the date of publication.

Research involving human participants, their data, or biological material

Policy information about studies with human participants or human data. See also policy information about sex, gender (identity/presentation), and sexual orientation and race, ethnicity and racism.

Reporting on sex and gender	n/a
Reporting on race, ethnicity, or other socially relevant groupings	n/a
Population characteristics	n/a
Recruitment	n/a
Ethics oversight	n/a

Note that full information on the approval of the study protocol must also be provided in the manuscript.

Field-specific reporting

Please select the one below that is the best fit for your research. If you are not sure, read the appropriate sections before making your selection.

☒ Life sciences ☐ Behavioural & social sciences ☐ Ecological, evolutionary & environmental sciences

For a reference copy of the document with all sections, see nature.com/documents/nr-reporting-summary-flat.pdf

Life sciences study design

All studies must disclose on these points even when the disclosure is negative.

Sample size	We did not perform formal power calculations prior to experimentation. For embryo experiments (Figs. 1–2), approximately 12 embryos were analyzed per 1°C interval across the full viable temperature range. Variability within temperature groups was low (Fig. 1C), and temperature trends were smooth. To avoid bias from unequal sampling density, we fitted scaling relationships to the median values per degree. Bootstrapping was used to estimate confidence intervals, confirming that sample sizes were sufficient. Extract experiments (Figs. 4–5) involved hundreds of oscillating droplets per experiment, spanning the entire temperature range. In vitro assays included replicate measurements across temperatures. Bootstrapping and curve fitting were applied consistently across all experimental systems to assess uncertainty.
Data exclusions	No data were excluded from the analyses.
Replication	All key findings were replicated across multiple experiments. Embryo experiments were performed on embryos obtained from different frogs, with separate experiments conducted at each temperature. Extract experiments were repeated across independently prepared extract batches to account for variability in extract quality and behavior. In vitro enzyme assays were replicated to ensure reproducibility of temperature-dependent trends.
Randomization	Embryos were randomly selected for each experiment, with each embryo set derived from a different frog. Frogs were randomly chosen from their tanks for egg collection and hormone injection. Each extract was prepared from independently collected eggs, with each batch potentially combining eggs from one or more frogs. Experiments were conducted independently and grouped post hoc based on experimental conditions (e.g., temperature, concentration of Morpholinos).
Blinding	Investigators were not blinded during sample preparation, data collection, or analysis. Given the nature of the experimental systems and the strong, consistent phenotypic readouts (e.g., cell cycle duration), blinding was not applicable to this study.

Behavioural & social sciences study design

All studies must disclose on these points even when the disclosure is negative.

Study description	n/a
Research sample	n/a
Sampling strategy	n/a
Data collection	n/a
Timing	n/a
Data exclusions	n/a
Non-participation	n/a
Randomization	n/a

Ecological, evolutionary & environmental sciences study design

All studies must disclose on these points even when the disclosure is negative.

Study description	n/a
Research sample	n/a
Sampling strategy	n/a
Data collection	n/a
Timing and spatial scale	n/a
Data exclusions	n/a
Reproducibility	n/a
Randomization	n/a
Blinding	n/a

Did the study involve field work? ☐ Yes ☐ No

Field work, collection and transport

Field conditions	n/a
Location	n/a
Access & import/export	n/a
Disturbance	n/a

Reporting for specific materials, systems and methods

We require information from authors about some types of materials, experimental systems and methods used in many studies. Here, indicate whether each material, system or method listed is relevant to your study. If you are not sure if a list item applies to your research, read the appropriate section before selecting a response.

Materials & experimental systems

n/a	Involved in the study
☐	☒ Antibodies
☒	☐ Eukaryotic cell lines
☒	☐ Palaeontology and archaeology
☐	☒ Animals and other organisms
☒	☐ Clinical data
☒	☐ Dual use research of concern
☒	☐ Plants

Methods

n/a	Involved in the study
☒	☐ ChIP-seq
☒	☐ Flow cytometry
☒	☐ MRI-based neuroimaging

Antibodies

Antibodies used

Western blots were blocked in 4% (w/v) non-fat dry milk in TBST and incubated overnight at 4°C with a 1:500 dilution of anti-cyclin B2 antibody (clone X121.10, mouse monoclonal IgG1κ, Santa Cruz Biotechnology, Cat. No. sc-53239, RRID:AB_831696). The antibody, provided at 200 µg/mL, is raised against Xenopus cyclin B2 and recommended for Western blot and immunoprecipitation of Xenopus samples. Blots were incubated for 1 hour at room temperature with a 1:10,000 dilution of HRP-conjugated anti-mouse IgG secondary antibody (GE Healthcare, Cat. No. NA931). Signal was detected using SuperSignal West Femto chemiluminescent substrate (Thermo Fisher Scientific).

Validation

The cyclin B2 antibody (X121.10) has been cited in six peer-reviewed publications and validated by the manufacturer in Xenopus egg extracts and XLK-WG cell lysates. It produces a single band at the expected molecular weight (~45kDa), consistent with cyclin B2 expression. We observed a comparable single band in our Xenopus extracts, supporting antibody specificity.

Animals and other research organisms

Policy information about studies involving animals; ARRIVE guidelines recommended for reporting animal research, and Sex and Gender in Research

Laboratory animals

Xenopus laevis (Nasco or Xenopus1); Xenopus tropicalis (Aquatic facility KU Leuven); and Danio rerio (Aquatic facility KU Leuven)

Wild animals

The study did not involve wild animals.

Reporting on sex

Sex was not considered for this study design.

Field-collected samples

This study did not include field-collected samples.

Ethics oversight

For the Xenopus laevis embryo experiments, the Ethical Committee for Animal Experimentation KU Leuven approved the frog handling, including the injections and egg collection, under Project 107-2021. All frog colonies were housed in recirculating systems with temperature and water quality regularly monitored.

For the extract experiments, all procedures were conducted in compliance with ethical regulations and the protocol approved by the Institutional Animal Care and Use Committee (IACUC) at the University of Michigan–Ann Arbor (PRO00011571).

For the Xenopus tropicalis embryo experiments, the Ethical Committee for Animal Experimentation KU Leuven approved frog handling procedures, including injections and egg collection, under Project 140/2014. The frogs were housed in recirculating systems in accordance with current regulations. Embryos were provided by the group of Prof. Dietmar Schmucker, who followed procedures equivalent to those described above, including the use of superovulation to stimulate embryo production. As only early cleavage stages were characterized—prior to stage 45—no separate ethical approval was required under Belgian legislation.

For Danio rerio (zebrafish), embryos were obtained from the KU Leuven zebrafish facility, which is recognized as an official stock center and authorized to distribute embryos. The fish were housed in recirculating systems in accordance with all applicable regulations. As only early cleavage stages—prior to 120 hours post-fertilization—were analyzed, no separate ethical approval was required under Belgian legislation.

Note that full information on the approval of the study protocol must also be provided in the manuscript.

Plants

Seed stocks	n/a
Novel plant genotypes	n/a
Authentication	n/a
